# Horizontal genome transfer by cell-to-cell travel of whole organelles

**DOI:** 10.1126/sciadv.abd8215

**Published:** 2021-01-01

**Authors:** Alexander P. Hertle, Benedikt Haberl, Ralph Bock

**Affiliations:** Max-Planck-Institut für Molekulare Pflanzenphysiologie, Am Mühlenberg 1, D-14476 Potsdam-Golm, Germany.

## Abstract

Recent work has revealed that both plants and animals transfer genomes between cells. In plants, horizontal transfer of entire plastid, mitochondrial, or nuclear genomes between species generates new combinations of nuclear and organellar genomes, or produces novel species that are allopolyploid. The mechanisms of genome transfer between cells are unknown. Here, we used grafting to identify the mechanisms involved in plastid genome transfer from plant to plant. We show that during proliferation of wound-induced callus, plastids dedifferentiate into small, highly motile, amoeboid organelles. Simultaneously, new intercellular connections emerge by localized cell wall disintegration, forming connective pores through which amoeboid plastids move into neighboring cells. Our work uncovers a pathway of organelle movement from cell to cell and provides a mechanistic framework for horizontal genome transfer.

## Teaser

This work elucidates the mechanisms of horizontal genome transfer and uncovers a new pathway of intercellular transport.

## INTRODUCTION

Natural grafts are widespread in plants (*1*–*3*), and the observation of natural grafting likely inspired the development of grafting techniques in agriculture and horticulture (*4*, *5*). Until recently, it was believed that, while proteins and RNA molecules can travel between scion and stock, no exchange of genetic information occurs across the graft junction. Genetic experiments have challenged this dogma by demonstrating that entire genomes migrate between scion and stock (*6*–*9*). All three genomes of the plant cell can engage in horizontal genome transfer between grafted plants. The process has great evolutionary significance in that it likely explains many cases of organelle capture (*10*–*13*) and provides a straightforward asexual mechanism for speciation by allopolyploidization (*9*). In animals and humans, cell-to-cell transfer of mitochondrial genomes restores the tumorigenic potential of cancer cells with dysfunctional mitochondria (*14*–*16*) and contributes to the recovery of neural tissue in the brain from stroke-induced damage (*17*).

How genomes are physically transferred from cell to cell and whether they travel as free DNA molecules or encapsulated in organelles is currently completely unknown. To elucidate the mechanism of horizontal genome transfer, we have studied the cellular events during formation of a graft union and attempted to observe cell-to-cell genome transfer in real time.

## RESULTS

### Organellar genome transfer across the graft junction

**Fig. 1:**
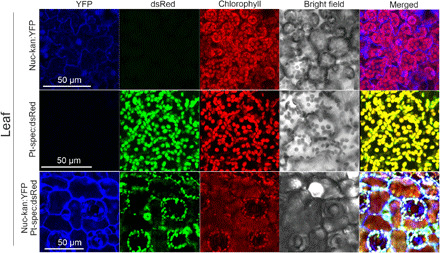
Detection of fluorescent markers by confocal laser scanning microscopy in leaf cells of plants used for grafting experiments (Nuc-kan:YFP and Pt-spec:dsRed) and a plant obtained from horizontal plastid genome transfer (bottom panel). Nuc-kan:YFP plants express the YFP reporter protein from their nuclear genome, whereas Pt-spec:dsRed plants express the dsRed reporter from their plastid genome (fig. S1A). YFP fluorescence is represented in blue and occurs in the nucleus and the cytosol. dsRed fluorescence is shown in green and, together with the red chlorophyll fluorescence, occurs exclusively in the plastids. Plants originating from horizontal plastid genome transfer show all three fluorescences (bottom panel).

Chloroplast DNA transfer across the graft junction can be detected genetically by using double selection for two compartment-specific selectable markers (*6*, *18*, *19*). To track the chloroplasts of scion and stock cells at the graft site and observe their possible movement, we performed reciprocal grafting experiments between a nuclear-transgenic tobacco line (Nuc-kan:YFP) and a transplastomic line (Pt-spec:dsRed). Nuc-kan:YFP plants harbor a kanamycin resistance gene and the gene for the yellow fluorescent protein (YFP) in their nuclear genome, while Pt-spec:dsRed plants express a spectinomycin resistance gene and the gene for the red fluorescent protein dsRed from their plastid genome (fig. S1A). The two fluorescent reporters, together with the chloroplast-specific chlorophyll fluorescence, allowed us to track the plastids and their potential movement between cells by confocal laser scanning microscopy (Fig. 1).

As expected, the YFP signal (shown in blue; Fig. 1) in Nuc-kan:YFP plants was detected in the nucleus and the cytosol, thus demarcating the cell borders. In transplastomic Pt-spec:dsRed plants, the dsRed fluorescence (shown in green) was detected exclusively in the plastids and, in green tissues, always colocalized with the chlorophyll fluorescence (shown in red; Fig. 1). In the absence of the respective fluorescent reporters, no fluorescence was detected in the dsRed and YFP channels, demonstrating that the two cell types can be unequivocally distinguished.

**Fig. 2:**
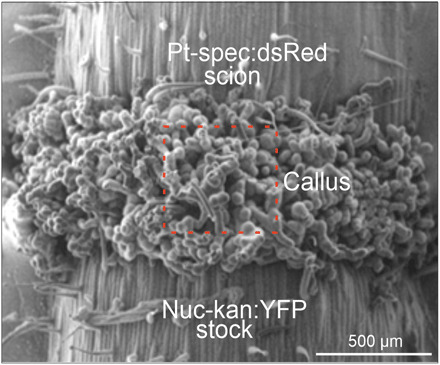
SEM image of a graft junction. Upon formation of the graft union, callus tissue proliferates from both stems, thus sealing the gap between stock and scion and reestablishing intercellular communication. Later, some callus cells differentiate into phloem and xylem, mediating vascular reconnection of scion and stock.

Next, we grafted Pt-spec:dsRed scions onto Nuc-kan:YFP stocks and vice versa to study the cellular events during graft union formation. After a few days, callus tissue had developed at the graft site, closing the wound and establishing a physical connection between the two graft partners (Fig. 2). Stem sections were then excised and assayed for gene flow between scion and stock by testing for the presence of cells that harbor both the kanamycin resistance gene (from Nuc-kan:YFP) and the spectinomycin resistance gene (from Pt-spec:dsRed; fig. S1B) by exposure to double selection on medium containing both antibiotics (*6*). Consistent with previous findings (*6*, *18*, *19*), this frequently yielded doubly resistant calli and regenerating shoots, indicative of horizontal transfer of plastid genomes (fig. S1, B and C). Horizontal genome transfer between scion and stock was confirmed by demonstrating that the regenerated plants show the expected expression and subcellular distribution of both fluorescent reporters (YFP and dsRed) in the same cell (Fig. 1).

**Fig. 3:**
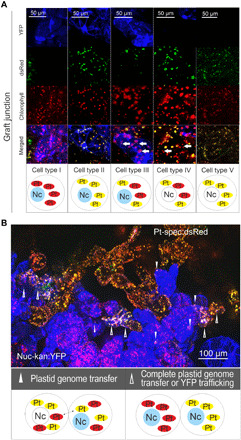
Intercellular exchange of plastid genomes during formation of the graft union. (**A**) Confocal images of callus cells within a (manually dissected) graft junction. Five different cell types can be distinguished. Types I and V represent Nuc-kan:YFP and Pt-spec:dsRed cells, respectively. Cells with a mixed population of plastids (types III and IV) originate from intercellular transfer of plastid genomes. Type II cells can arise through intercellular genome transfer but could alternatively represent YFP (protein or mRNA) exchange between neighboring cells. See text for details. Arrows mark transferred plastids. Note that transferred plastids are smaller than resident plastids (table S1). Nc, nucleus; Pt, plastid. (**B**) Callus cells forming a graft union. Within a single graft junction, multiple events of plastid genome exchange (▲) are detected, in addition to a number of events that represent either complete plastid genome exchange (i.e., complete replacement of the plastid population of the recipient cell) or macromolecular trafficking of the YFP (∆).

While these and previous data (*6*, *18*, *20*) provide clear genetic evidence of horizontal genome transfer across the graft junction, they cannot distinguish between transfer of free plastid genome molecules and transfer of organelles with the genomes enclosed and also do not provide information about the cellular processes involved. To shed light on the underlying mechanism, we analyzed grafts at various stages of tissue reunion and doubly resistant calli emerging from the graft junction by confocal microscopy. To this end, graft unions and calli were manually dissected for live-cell imaging. Within growing calli, cells harboring plastids with transferred genomes were readily distinguishable from the cells of the two graft partners (Fig. 3A). Within the callus tissue, five cell types could be distinguished on the basis of their combination of organellar and nuclear fluorescence markers. Cells of the two graft partners were recognized by their exclusive expression of either YFP in the nucleus and the cytosol (cell type I) or dsRed in all plastids (cell type V). Cells with mixed populations of wild-type plastids and dsRed-expressing plastids either displayed YFP expression (indicative of plastid genome transfer from Pt-spec:dsRed to Nuc-kan:YFP cells; cell type III) or did not show it (indicative of plastid genome transfer from Nuc-kan:YFP to Pt-spec:dsRed cells; cell type IV). Last, cells showing YFP fluorescence and harboring a homogeneous population of dsRed-expressing plastids were also observed (cell type II). While these cells could represent plastid genome transfer events in which the transferred transplastomic genome has completely replaced the resident wild-type plastid genome, they could also result from macromolecular trafficking of YFP mRNA or protein from a neighboring Nuc-kan:YFP cell into a Pt-spec:dsRed cell. Cell-to-cell transport of (nucleus-encoded) cytosolic green fluorescent protein (GFP) through plasmodesmata is known to occur (*21*, *22*), and therefore, type II cells cannot be unambiguously classified as genome transfer events. By contrast, plastid-expressed reporter proteins remain contained within the plastid and thus allow the clear identification of transfer events.

Type II cells are relatively frequent and occur in approximately 1 of 10 contacting cells, presumably mainly due to the uptake of cytosolic YFP protein from Nuc-kan:YFP cells by neighboring Pt-spec:dsRed cells (through cell wall openings and/or secondary plasmodesmata). Type III and IV events are less frequent and, on the basis of the presence of dsRed-labeled plastids in Nuc-kan:YFP cells, have been estimated to occur in approximately 1 of 20 to 40 contacting cells. Thus, within a single graft union, multiple events of intercellular exchange of plastid genomes are observed (Fig. 3B), indicating that cell-to-cell genome transfer occurs at high frequency. Our data also demonstrate that genome transfer occurs bidirectionally, as evidenced by identification of both cell types III and IV. Last, our microscopic investigations revealed that transfer events can occur before vascular reconnection of the grafted tissues and are already observed when callus cells emerging from stock and scion adhere to each other (*23*, *24*).

### Cell wall pores facilitate genome travel between cells

**Fig. 4:**
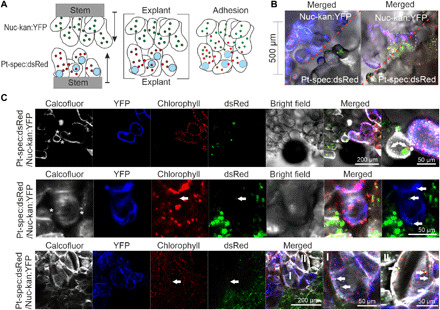
Plastid mixing in grafted callus cells. (**A**) Callus grafting. Callus tissue was obtained by placing stem sections from Nuc-kan:YFP and Pt-spec:dsRed plants on regeneration medium. Tissue proliferating from the cambium ring was grafted by aligning callus explants with a micromanipulator and placing them between agarose blocks. Continued growth results in cell adhesion and tissue fusion. (**B**) Confocal images (with YFP and dsRed fluorescences merged) of the graft junction before (left) and after (right) cell adhesion. Nuc-kan:YFP cells contain plastid genomes acquired from Pt-spec:dsRed (white dashed box). The red dashed line indicates the border between the grafted tissues. (**C**) Images of individual callus grafts with cells containing a mixed population of plastids. The top panel shows an event of plastid genome transfer from a Nuc-kan:YFP cell into a Pt-spec:dsRed cell. The two bottom panels show transfer events from Pt-spec:dsRed into Nuc-kan:YFP cells. Note the initially small size of the acquired plastids (arrows) within the adhered callus cells (top and middle panels). They later regain normal size (bottom panel). (I) and (II) indicate two independent transfer events in adjacent cells. Costaining of cell walls reveals protrusions of adhered callus cells (asterisks in top and middle panels) and irregular staining of the cell walls after establishment of the graft union (bottom).

To simplify the microscopic study of the cellular events leading to horizontal genome transfer, we established an experimental setup based on grafting of calli (*25*) rather than whole plants (Fig. 4A). Callus tissue induced from stem sections of Nuc-kan:YFP and Pt-spec:dsRed plants was placed between agar blocks and cultivated until tissue fusion had occurred (Fig. 4B). Cells at the border of the two tissue types were then analyzed for possible events of genome transfer by live-cell confocal microscopy. To visualize the cell walls separating the different cell types, the grafted callus tissue was additionally stained with the fluorescent dye calcofluor white (Fig. 4C).

Both Nuc-kan:YFP cells and Pt-spec:dsRed cells were frequently found to contain plastids and/or plastid genomes acquired from neighboring cells at the graft junction (Fig. 4, B and C). The newly acquired type of plastids was consistently smaller than the resident plastids (table S1). As tissue organization and establishment of the graft union progressed, the plastids grew and reached normal size, indicating their full integration into the resident population of organelles (Fig. 4C, bottom).

Staining of cell walls revealed pronounced protrusions between connecting callus cells (Fig. 4C, top). Together with the irregular and patchy staining of cell walls between adhering cells (Fig. 4C, bottom), this observation suggested that substantial rearrangements of cell wall architecture occur upon formation of the graft union, raising the possibility that these structural changes promote genome or organelle exchange between cells.

**Fig. 5:**
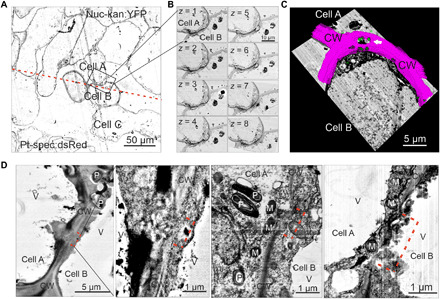
Cell wall pores allow exchange of large cellular structures between callus cells. (**A**) Overview electron microscopy image (S^3^EM) of a cryo-fixed callus assembly containing cells with transferred plastid genomes. The black box indicates a pair of adhered cells at the graft junction; the red dotted line marks the border between the grafted tissues. (**B**) BSE images of eight consecutive 70-nm-thick sections (z-stack) spanning the cell wall contact zone of two adhered callus cells [within the boxed area in (A)]. The middle lamella between the cells identifies them as adhered cells (cells A and B) rather than daughter cells. By contrast, no middle lamella is present between cell B and cell C [in (A)]. (**C**) Tomogram of a cell wall (magenta) between adhered callus cells. The tomogram reveals a direct cytoplasmic connection (arrow) and organelles (green) that are small enough for direct passage. (**D**) Individual cell wall openings between adhered callus cells (marked by red dotted lines) identified by electron microscopy. M, mitochondrion; V, vacuole; CW, cell wall.

Our simplified experimental setup (Fig. 4A) also allowed direct cryofixation of the grafted tissue (without any previous manipulation), thus facilitating the ultrastructural analysis of adhering callus cells (Fig. 5A). We dissected the cell wall contact zones in the graft union by serial sectioning scanning electron microscopy (S^3^EM) and used images of consecutive sections for tomographic reconstructions (Fig. 5, B and C). The tomograms (Fig. 5C) and electron microscopic images (Fig. 5D) revealed cytoplasmic connections across the cell wall between Nuc-kan:YFP and Pt-spec:dsRed cells (Fig. 5C). The organelles present in the vicinity of these cell wall pores were smaller than the pores (which had a diameter of up to 1.5 μm; Fig. 5, B to D, and table S1), raising the possibility that entire plastids move through the pores from cell to cell. Plastids and mitochondria were frequently seen at or even within the pores (Fig. 5D, two right images, and fig. S2).

### Cytoplasmic protrusions support organelle exchange between cells

**Fig. 6:**
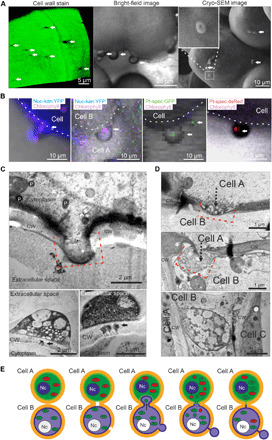
Budding callus cells in graft unions. (**A**) Confocal image of a cell wall at the graft junction. Staining with calcofluor white shows open pores (left). In addition, bud-like protrusions emerge from the cell surface (middle panel), which are also seen by cryo-SEM (right). The structures collapse under high vacuum, but the central pore remains visible (inset). (**B**) Confocal images of the bud-like protrusions. In Nuc-kan:YFP lines, the cytosolic YFP signal extends into the buds (right two images, blue signal). Chlorophyll fluorescence is detectable within some buds, indicating the presence of plastids (magenta signal). The presence of plastids (white arrows) was confirmed with transplastomic lines expressing GFP (*6*) or dsRed (left two images). (**C**) Analysis of the bud-like protrusions by TEM of cryo-fixed cells. The buds emerged from cell wall pores with an approximate width of 1.5 μm. Some buds keep narrow connections to their mother cell (black arrows). (**D**) Ultrastructural analysis of buds reaching into neighboring cells. The buds are rich in cytosol and organelles. The red dashed lines in (C) and (D) mark the openings in the cell walls of adhered callus cells. (**E**) Schematic representation of the cellular events described in (A) to (D).

To shed light on the mechanisms of intercellular pore formation, we investigated the walls of callus cells in graft unions by confocal and electron microscopy. For confocal microscopy, the cell walls of proliferating callus cells were stained with calcofluor white (Fig. 6A and fig. S3A) and monitored for 4 days. While up to the second day the walls showed homogeneous surface staining, numerous holes (visible as unstained areas) started to appear at day 3 (fig. S3A). The holes formed in the walls of surface-exposed callus cells as well as in the walls between neighboring adhered cells (fig. S3B). Through these holes, bud-like protrusions emerged from the cells, which were also observed by light microscopy and cryo–scanning electron microscopy (cryo-SEM) (Fig. 6A and fig. S3). When the structures collapsed under high vacuum, a central pore remained visible (Fig. 6A, inset), supporting a function of the cell wall holes as outlet ports for the buds. The number of buds increased following transfer of calli to fresh growth medium (fig. S3D), suggesting that expanding cytosol squeezes through the pores. Consistent with this interpretation, the presence of cytosolic YFP and organelles was detected by confocal microscopy (Fig. 6B). The presence of plastids was evidenced by detection of both chlorophyll fluorescence and fluorescent proteins (dsRed and GFP) expressed from the plastid genome (Fig. 6B and fig. S3C). In contrast to dsRed and GFP fluorescence, chlorophyll fluorescence was often relatively weak, indicating low amounts of chlorophyll and low photosynthetic activity of the extruded plastids.

Buds filled with extruded cytoplasmic material were also readily detected by transmission electron microscopy (TEM) imaging of cryo-fixed tissue (Fig. 6, C and D). The buds emerged from cell wall pores with an approximate diameter of 1.5 μm (Figs. 5, B to D, and 6, C and D) and reached into the cytosol of the neighboring cell (Fig. 6, D and E), suggesting formation of intercellular connections that allow organelle transfer from cell to cell. Subsequent wall closure by resumed cell wall synthesis resulted in buds virtually pinching off the mother cell. However, analysis of consecutive sections revealed that they remained connected with the mother cell by thin channels (Fig. 6, C and E) that, based on their structure and diameter, may represent complex secondary plasmodesmata (*26*).

### Plastid dedifferentiation into highly mobile amoeboid plastids

**Fig. 7:**
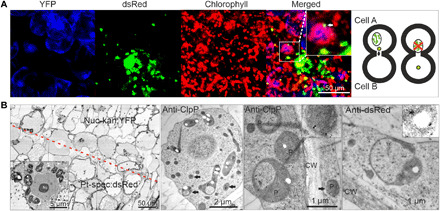
Plastids of callus cells in the graft union dedifferentiate into amoeboid proplastids. (**A**) Confocal microscopy images of cells harboring transferred plastids. Whereas plastids newly acquired from a neighboring donor cell have a diameter of approximately 1 μm, resident plastids in the recipient cells have a diameter of approximately 5 μm. Only the small plastids are likely able to pass through the cell wall pores with an estimated size exclusion limit of 1.5 μm (see Figs. 5, B to D, and 6, C and D). (**B**) Callus cells contain a mixed population of morphologically distinguishable plastids. Starch granule-containing amyloplast-like plastids represent the main plastid type. The inset in the right image shows a starch granule (white) at high magnification. Immunoelectron microscopy of cryo-fixed samples (using anti-ClpP and anti-dsRed antibodies) additionally identified small rod-like amoeboid organelles and proplastid-like small spherical or bean-shaped organelles (black arrows and inset in the second image from the right) as plastids. Most of these small plastids contain few or no internal membranes and lack starch granules.

Confocal microscopy images of cells that had received plastid genomes from neighboring cells at the graft junction revealed that the “alien” plastids were substantially smaller than the resident plastids (Fig. 7A and table S1). Having a diameter of approximately 1 μm, these small plastids fit through the pores observed in cell walls of connecting cells at the graft junction, whereas normal-sized chloroplasts do not (Figs. 5, C and D, and 7A). The low amount of chlorophyll detected in these plastids indicates that the size reduction largely occurs at the expense of the internal membrane system, the thylakoids harboring the photosynthetic apparatus (Figs. 3A, 4C, and 6B).

To investigate whether dedifferentiation of plastids facilitates transfer of entire organelles (with their genomes encapsulated), we analyzed the population of plastids in callus cells at the graft junction by electron microscopy (Fig. 7B). The predominantly present plastids harbored big starch granules and were largely devoid of internal thylakoid membranes (Fig. 7B, left image). In addition, we observed rod-like amoeboid plastids and proplastid-like small spherical or bean-shaped plastids with diameters as small as 200 nm (Fig. 7B, black arrows, and fig. S4). These small organelles were unambiguously identified as plastids by immunoelectron microscopy with plastid-specific antibodies (Fig. 7B and fig. S4B). Because these plastids largely lacked thylakoids, antibodies against stromal proteins were used. Antibodies against ClpP, a subunit of the Clp protease in the chloroplast, recognized the small proplastid-like organelles with high specificity (Fig. 7B and fig. S4B). The identity of the organelles was further confirmed with anti-RbcL and anti-GLN2 antibodies (recognizing the large subunit of Rubisco and the plastid glutamine synthetase, respectively; fig. S4B). Last, dsRed-specific antibodies also recognized the amoeboid and spherical organelles in Pt-spec:dsRed cells as plastids (Fig. 7B).

**Fig. 8:**
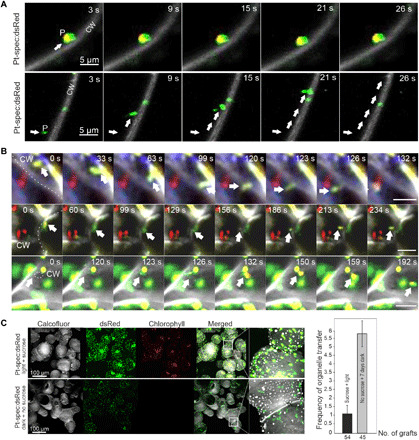
Dedifferentiated plastids are highly mobile and move from cell to cell. (**A**) Dedifferentiated small and amoeboid plastids show increased mobility. Although normal plastids remain at their position within the cell for a long time (top series), amoeboid and spherical plastids move rapidly inside the cell (bottom series), often along the plasma membrane (see movies S1 and S2). (**B**) Transfer of dedifferentiated plastids between living cells in real time. The mobile plastids are marked by white arrows; the cell wall crossed by them is represented as dotted line (top and middle panels). A cell wall pore through which a plastid travels is marked by the dotted circle (bottom). See also movies S3 to S5. (**C**) Plastid dedifferentiation facilitates intercellular transfer. When plastid dedifferentiation is induced by keeping callus tissue in the dark for 3 days in the absence of an external carbon source, loss of chlorophyll fluorescence occurs, indicating thylakoid degradation. In parallel, plastid size and shape markedly change. Experimental stimulation of dedifferentiation increases the frequency of intercellular organelle transfer across the graft junction more than fivefold. The *x* axis represents the number of successful grafts analyzed; the *y* axis represents the number of doubly resistant calli obtained per graft.

To further test the idea that the dedifferentiated small plastids move from cell to cell, their mobility was analyzed and compared to that of normal plastids. Although the normal-sized plastids (with a diameter of ~5 μm) were largely stationary and showed only moderate Brownian-like movements, the small spherical (diameter ≤ 1 μm) and amoeboid plastids were found to be highly mobile (Fig. 8A, table S1, and movies S1 and S2).

### Observation of cell-to-cell movement of plastids in real time

To provide ultimate proof of organelle movement from cell to cell, we next attempted to observe the intercellular transfer of plastids in real time. To this end, we followed the movements of mobile dedifferentiated plastids in cells at the graft junction. Multiple events of cell-to-cell migration of amoeboid plastids were observed (Fig. 8B and movies S3 to S5). Analysis of fluorescent markers confirmed that the organelle transfer occurred across the graft junction, between Pt-spec:dsRed and Nuc-kan:YFP cells (Fig. 8B and movies S3 and S4). These findings unambiguously demonstrate that genomes move horizontally in graft unions via organelle travel from cell to cell.

Last, we wanted to confirm that plastid motility and dedifferentiation promote intercellular organelle transfer. To this end, we tested various growth conditions for their impact on plastid motility. We found that dark-induced carbon starvation stimulates dedifferentiation into small and amoeboid plastid types and also substantially increases organelle motility (Fig. 8C, table S1, and movies S1 and S2). We then used double selection for the nuclear and plastid resistance genes (*6*, *18*) to genetically quantify horizontal genome transfer (fig. S1 and Fig. 8C). In comparison to grafts fed with sucrose and incubated in the light, carbon starvation (i.e., absence of an exogenously supplied carbon source) resulted in a more than fivefold increase in the frequency of horizontal genome transfer events (Fig. 8C). This finding indicates that plastid dedifferentiation (and the concomitant size reduction and increased mobility) facilitates intercellular organelle transfer.

### Transferred dedifferentiated plastids contain DNA

Chloroplast stroma can be routed by autophagy into the vacuole via spherical bodies dubbed Rubisco-containing bodies (*27*, *28*). In addition, plastids can produce stroma-filled extensions (referred to as stromules) that are highly dynamic and can break off or be removed by the autophagic machinery (*29*–*32*). As stromules do not contain chloroplast DNA (*33*), they are unlikely to serve as DNA vehicles in horizontal genome transfer. We, therefore, wanted to exclude the possibility that the dedifferentiated amoeboid plastids we had observed to travel from cell to cell represent fragmented plastids or stromule-derived structures that are devoid of plastid DNA. To this end, we stained cells harboring dedifferentiated mobile plastids with the DNA-intercalating fluorescent dye SYBR Green.

**Fig. 9:**
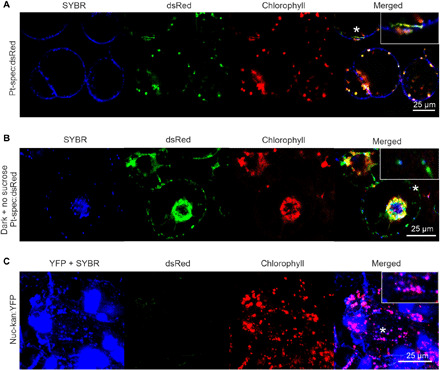
Dedifferentiated plastids of callus cells in the graft union contain DNA. Staining of DNA in callus cell plastids. (**A**) Detection of DNA in plastids by colocalization of the fluorescences of (i) the DNA-intercalating dye SYBR Green, (ii) the plastid-expressed dsRed protein, and (iii) the chloroplast-specific pigment chlorophyll in Pt-spec:dsRed callus cells via confocal laser scanning microscopy. (**B**) Detection of DNA in dedifferentiated plastids. Plastid dedifferentiation in Pt-spec:dsRed callus cells was induced by carbon starvation (growth in the dark and in the absence of sucrose), followed by staining of DNA with SYBR Green. Colocalization of SYBR Green fluorescence (blue) with plastid-specific dsRed fluorescence (green) confirms the presence of DNA in dedifferentiated plastids. (**C**) Detection of plastid DNA in Nuc-kan:YFP cells. Although SYBR Green and YFP fluorescence strongly overlap (YFP + SYBR), colocalization of SYBR Green fluorescence (green) and chlorophyll fluorescence (red) in dedifferentiated plastids of Nuc-kan:YFP cells can be seen and confirms the presence of DNA. The asterisks denote the plastids that are shown enlarged in the insets.

We first stained Pt-spec:dsRed callus cells with SYBR Green to unequivocally distinguish between the SYBR Green and dsRed fluorescence (Fig. 9A). Next, we stained callus cells under carbon starvation conditions with SYBR Green and confirmed the presence of DNA in dedifferentiated motile plastids by colocalization of SYBR Green and dsRed fluorescence (Fig. 9B). Because YFP and SYBR Green show largely overlapping fluorescence excitation and emission patterns, the detection of DNA in plastids of Nuc-kan:YFP cells is more challenging. However, localization of DNA within plastids (above the background of the YFP fluorescence) can also be seen in Nuc-kan:YFP cells (Fig. 9C).

**Fig. 10:**
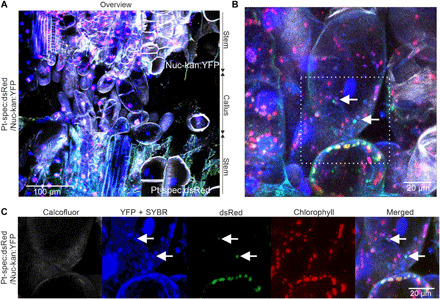
Transferred plastids contain DNA. Confocal images of callus cells within a (manually dissected) Pt-spec:dsRed/Nuc-kan:YFP graft junction. (**A**) Overview image of the graft union, with the cell walls stained with calcofluor white and the (nuclear and organellar) DNA visualized with SYBR Green. In Nuc-kan:YFP cells, SYBR Green fluorescence is detected in the nucleus and the organelles, together with the YFP fluorescence in the nucleus and the cytosol (YFP + SYBR). Chloroplasts are recognized by their red chlorophyll fluorescence. In Pt-spec:dsRed cells, DNA in the nucleus and the organelles is visualized by SYBR Green staining. In addition, the plastids show chlorophyll fluorescence and dsRed fluorescence. (**B**) Among the callus cells, cells with mixed populations of plastids represent events of organelle transfer across the graft junction. (**C**) Enlargement of the region boxed in (B) (white dotted square). Note that transferred plastids are smaller than resident plastids and contain DNA, as evident from colocalization of dsRed and SYBR Green fluorescence (arrows).

To ultimately confirm that plastid genomes are transferred across graft junctions by cell-to-cell travel of dedifferentiated plastids, we applied SYBR Green staining to junctions of grafted Nuc-kan:YFP and Pt-spec:dsRed plants (Fig. 10A). Plastid transfer events were identified by the detection of cells harboring dedifferentiated Pt-spec:dsRed plastids within Nuc-kan:YFP cells (Fig. 10B). Colocalization of SYBR Green fluorescence and dsRed fluorescence in these plastids (Fig. 10C) unambiguously demonstrated that the transferred plastids contain DNA.

## DISCUSSION

Genetic data revealed that nuclear, plastid, and mitochondrial genomes are asexually transferred between cells and organisms of the same or different species (*6*–*9*, *14*, *15*, *17*). In plants, horizontal transfer of plastid or mitochondrial genomes results in plants with new combinations of nuclear and organellar genomes, while the horizontal transfer of nuclear genomes generates new plant species that are allopolyploid (*9*). In this work, we have elucidated the cellular mechanisms underlying the horizontal transfer of plastid genomes. Compared to nuclei and mitochondria, plastids offer several advantages for studies into the mechanisms of horizontal genome transfer. First, unlike mitochondria, plastids do not normally fuse and recombine, thus making it possible to follow the fate of individual organelles. Second, plastids can be genetically transformed, facilitating fluorescent protein expression within the organelle. Last, compared to the nucleus from which fluorescent reporter proteins usually leak out into the cytosol [and would potentially be taken up by a horizontally acquired second nucleus (*7*)], plastid-expressed reporter proteins remain contained within the plastid, thus making organelle labeling unambiguous.

The research reported here demonstrates that the horizontal genome transfer occurs by cell-to-cell transfer of entire organelles, with the genomes encapsulated within them. Our microscopic investigations have uncovered a remarkable series of events involved in the movement of plastids between plants across a graft junction. The initial formation of undifferentiated callus tissue is followed by conspicuous changes in cell wall structure, most notably the formation of large pores. Cytoplasmic material passes through these pores, ultimately connecting neighboring cells and facilitating the passage of large cellular structures. At the same time, plastids undergo marked changes in their morphology and become highly mobile (Fig. 8A and movies S1 and S2). Dedifferentiation and enhanced mobility are likely promoted further by induction of a local starvation response, which, upon (natural or experimental) grafting, may be an immediate consequence of wounding and severing of vascular bundles. We could observe the passage of plastids from cell to cell and across the graft junction in real time (Fig. 8B and movies S3 to S5), ultimately confirming that organelles as large as entire plastids can travel between cells.

At present, we can only speculate about possible physiological functions of the pores forming in the cell walls of neighboring cells. Pore formation could be the initiating step in the biogenesis of secondary plasmodesmata (*26*, *34*) or, alternatively, could be part of a starvation response in that the pores facilitate the exchange of nutrients between stock and scion before vascular reconnection and formation of secondary plasmodesmata at the graft junction.

While in previous reports antibiotic selection was used to detect events of horizontal genome transfer, selection was not used in any of the microscopic investigations of graft unions in our present study. This fact is important in that it demonstrates that high-frequency plastid movement from cell to cell occurs also in the absence of selection for genome transfer.

The initial horizontal genome transfer events are restricted to cells in the graft union. However, the events can readily become heritable through lateral shoot initiation from the graft site. As grafting involves wounding and wounding induces local phytohormone synthesis (*24*, *35*, *36*), outgrowth of lateral shoots is a common phenomenon in both natural and man-made grafts (*7*).

Although plastids are often stereotypically portrayed as lentil-like ellipsoid organelles, a wide range of plastid types and associated morphologies have been described (*37*). In addition to a variety of shapes that especially nongreen plastid types can adopt (*37*, *38*), plastids can also form protrusions (stromules) that are highly dynamic and additionally affect plastid morphology (and mobility) through their interaction with the cytoskeleton (*30*, *31*, *39*–*41*). Our observation that plastids in callus cells at graft unions adopt a variety of alternative shapes and, moreover, become highly mobile is consistent with the view that, at least under certain conditions, plastid morphology and behavior are highly dynamic.

In both plants and animals, the ability of tissues to graft represents an active developmental program. In seed plants, it involves (i) callus growth at the graft site, (ii) establishment of new vascular connections between scion and stock, and (iii) de novo generation of intercellular connections by formation of secondary plasmodesmata (*5*, *26*, *42*). The latter requires local removal of the cell wall (*26*) and, together with induction of callus growth, may be directly involved in facilitating organelle mobility. Although thus far horizontal genome transfer has been shown only in the context of grafting, secondary plasmodesmata are also frequently inserted into existing cell walls between nondividing cells in intact plant tissues (*34*). Whether cell-to-cell movement of organelles also occurs upon formation of secondary plasmodesmata in leaves, flowers, and other tissues will be interesting to investigate in the future.

It seems possible that the horizontal transfer of nuclear and mitochondrial genomes uses similar cellular mechanisms as found here for plastids. However, the involvement of alternative (or additional) mechanisms cannot be ruled out at present. For example, partial cell fusion (e.g., fusion of a cell bud with a neighboring cell in the graft junction) could also transfer organelles from one cell to another and might be an attractive possibility to investigate, especially for the horizontal movement of nuclei (that are much larger than the plastids we observed to travel from cell to cell in this work). The abovementioned technical obstacles make it challenging to investigate the mechanism of horizontal genome transfer in nuclei and mitochondria.

In addition to grafts, horizontal DNA transfer was also shown to occur between parasitic plants and their host plants (*13*, *43*, *44*). In this regard, it is important to realize that the establishment of haustorial connections between parasite and host is mechanistically very similar to grafting (*45*), making it likely that similar cellular mechanisms as described here are also involved in horizontal DNA transfer processes mediated by plant-plant parasitism.

In summary, the findings reported here have elucidated the cellular mechanisms that underlie horizontal genome transfer events in plant grafts and uncovered a previously unknown pathway of intercellular transport by which very large cellular structures (including entire plastids) are exchanged between cells.

## MATERIALS AND METHODS

### Plant material

Sterile tobacco (*Nicotiana tabacum* cv. Petit Havana and cv. Samsun NN) plants were grown on agar-solidified synthetic medium containing sucrose (30 g/liter) (*46*). Homoplasmic plastid-transformed (transplastomic) Pt-spec:dsRed plants were provided by S. Schillberg (Fraunhofer IME, Aachen) and carry a dsRed expression cassette (driven by the ribosomal RNA operon promoter and a chimeric 5′ untranslated region from the chloroplast *psbA* and *clpP* genes) that was inserted as a dicistronic operon with the selectable marker gene *aadA* (*47*) into the intergenic region between the *rpl32* and *trnL* genes in the tobacco plastid genome (fig. S1A). Nuclear transgenic Nuc-kan:YFP lines harbor a YFP expression cassette under the control of the CaMV 35S promoter and terminator (*6*). Nuc-kan:YFP plants are kanamycin resistant and show YFP accumulation in the cytosol and the nucleus, while Pt-spec:dsRed plants are spectinomycin resistant and show dsRed accumulation in chloroplasts.

### Grafting and selection for intercellular genome transfer

To exclude the influence of pathogens or endophytes, grafting experiments were performed with plants raised under aseptic conditions as described previously (*6*, *7*, *18*). Stock and scion were allowed to fuse, followed by either microscopic analysis or selection for genome transfer by exposure of excised graft sites to double selection on a plant regeneration medium containing spectinomycin (500 mg/liter) and kanamycin (250 mg/liter) (*6*). As controls, stem cuttings and leaf explants from Nuc-kan:YFP and Pt-spec:dsRed plants raised under identical conditions were exposed to the same medium. Doubly resistant calli and shoots were transferred to fresh plates and regenerated again under antibiotic selection to isolate homoplasmic genome transfer lines (*18*, *19*). Regenerated shoots were rooted on hormone-free medium, transferred to soil, and grown to maturity under standard greenhouse conditions.

### Callus grafting

For grafting of callus tissue, internodes of sterile tobacco plants (with a stem diameter of approximately 3 to 5 mm) were cut into 1- to 2-mm-thick discs and incubated on regeneration medium for 3 days. Callus tissue proliferating from the cambial ring was collected and used for grafting. Callus grafting was performed by placing callus samples together in a narrow gap between agar-solidified synthetic medium suitable for direct confocal imaging or directly into aluminum carriers suitable for high-pressure freezing. In an alternative approach, stem discs were grafted using a similar setup. The cells at the interface between the tissues typically adhered within 24 hours. For the isolation of stable horizontal genome transfer events, grafted callus tissue was transferred to double selection as described previously (*6*).

### Confocal laser scanning microscopy

Subcellular localization of dsRed and YFP fluorescence was determined by confocal laser scanning microscopy (TCS SP5 or TCS SP8, Leica, Wetzlar, Germany) using an argon laser for excitation (512 nm) and a 520- to 560-nm filter for detection of YFP fluorescence, a diode-pumped solid state laser at 561 nm for excitation, and a 575- to 603-nm filter for detection of dsRed fluorescence. Chlorophyll fluorescence was monitored by excitation with a helium-neon laser at 633 nm or a diode laser at 405 nm and detection with a 650- to 700-nm filter.

### In vivo cell wall staining

For staining of cell walls, callus tissue was immersed in 0.01% (w/v) calcofluor (Fluorescent Brightener 28) dissolved in water and stained for 10 min. Subsequently, the samples were rinsed twice with water and immediately used for imaging. Calcofluor was excited with a 405-nm diode laser, and fluorescence was detected at 415 to 480 nm.

### In vivo DNA staining

For staining of DNA, callus samples were immersed in a 0.01% solution of SYBR Green and stained for 10 min. Subsequently, the samples were rinsed twice with water for 5 min and then used for imaging. SYBR Green was excited using an argon laser at 488 nm, and fluorescence was detected at 500 to 550 nm.

### High-pressure freezing and freeze substitution

Tissue samples were high-pressure–frozen using a Leica HPM100 instrument. For ultrastructural analysis, samples were freeze-substituted in 1% OsO_4_ and 0.1% uranyl acetate in acetone. For immunolabeling, samples were freeze-substituted in 0.5% uranyl acetate in acetone and embedded into LR White medium at −20°C. Subsequently, samples were ultraviolet-polymerized at −20°C.

### Transmission electron microscopy

To prepare samples for TEM analysis, tissue sections were cut using a Leica UC-6 ultramicrotome. For contrasting, sections were poststained with methanolic uranyl acetate (2% in 50% methanol) for 30 min, followed by a 10-min incubation in lead citrate (Reynolds’ stain). Images were acquired with a Zeiss EM 912 Omega TEM (Carl Zeiss, Oberkochen, Germany).

### Environmental scanning electron microscopy

Graft junctions were plunged into liquid nitrogen and then transferred into the sample chamber of a tabletop environmental SEM instrument (Hitachi TM3030Plus). Images were acquired at 5 kV using a secondary electron detector under high vacuum.

### Serial sectioning scanning electron microscopy

For S^3^EM analysis, adhered callus cells in embedded cell assemblies were cut into ribbons of consecutive 70-nm-thin sections using a histo knife. The ribbons were then transferred onto a coverslip, dried at 70°C for at least 5 min, and contrasted. Subsequently, the samples were mounted onto stubs and imaged using a JEOL 7500F SEM at 15 kV and a probe current of 10 pA at a working distance of 8 mm using a backscattered electron (BSE) detector. Tomographic reconstructions from acquired images were obtained using the Fiji image processing package TrakEM2.

### Immunogold labeling for electron microscopy

For immunogold labeling, cryo-fixed and freeze-substituted samples embedded into LR White were cut into 100-nm-thin sections. Nonspecific antibody binding was reduced by incubation of samples in blocking buffer [phosphate-buffered saline–Tween 20 (PBST) containing 2% bovine serum albumin and 0.1% fish gelatin; Sigma-Aldrich] for 1 hour at room temperature. Antigens were detected by incubation in blocking buffer containing anti-dsRed (1:100 dilution, mouse; ChromoTek GmbH, Planegg-Martinsried, Germany), anti-ClpP (1:200, rabbit; provided by A. Clarke, University of Gothenburg, Sweden), anti-RbcL (1:200, rabbit; Agrisera, Vännäs, Sweden), or anti-GLN2 (1:200, rabbit; Agrisera) antibodies for 1 hour at room temperature. Excess antibodies were removed by six rinses with PBST buffer for 3 min each. Following hybridization to sections, bound primary antibodies were detected by incubation for 1 hour in blocking buffer containing 10 nm (for mouse; Cell Signaling Technology, Danvers, MA) or 25 nm colloidal gold-labeled secondary antibodies (goat anti-rabbit antibody, 1:20; AURION, Wageningen, The Netherlands). After six rinses with PBST and three rinses with double-distilled water for 3 min each, samples were contrasted with aqueous 2% uranyl acetate for 12 min and Reynolds’ lead citrate for 7 min.

## Supplementary Material

http://advances.sciencemag.org/cgi/content/full/7/1/eabd8215/DC1

Video S1

Video S2

Video S3

Video S4

Video S5

Adobe PDF - abd8215_SM.pdf

Horizontal genome transfer by cell-to-cell travel of whole organelles

## SUPPLEMENTARY MATERIALS

Supplementary material for this article is available at http://advances.sciencemag.org/cgi/content/full/7/1/eabd8215/DC1

## Appendix

View/request a protocol for this paper from *Bio-protocol*.

## References

[1] Seidel C. F., Ueber Verwachsungen von Stammen und Zweigen von Holzgewächsen und ihren Einfluss auf das Dickenwachsthum der betreffenden Theile. Naturwiss. Ges. Isis Dresden Sitzber. 1879, 161 (1879).

[2] Küster E., Über Stammverwachsungen. Jahrb. Wiss. Bot. 33, 487–512 (1899).

[3] Beddie A. D., Natural root grafts in New Zealand trees. Trans. Proc. R. Soc. 71, 199 (1942).

[4] Mudge K., Janick J., Scofield S., Goldschmidt E. E., A history of grafting. Hortic. Rev. 35, 437–493 (2009).

[5] Goldschmidt E. E., Plant grafting: new mechanisms, evolutionary implications. Front. Plant Sci. 5, 727 (2014). 25566298 10.3389/fpls.2014.00727 PMC4269114

[6] Stegemann S., Bock R., Exchange of genetic material between cells in plant tissue grafts. Science 324, 649–651 (2009). 19407205 10.1126/science.1170397

[7] Fuentes I., Stegemann S., Golczyk H., Karcher D., Bock R., Horizontal genome transfer as an asexual path to the formation of new species. Nature 511, 232–235 (2014). 24909992 10.1038/nature13291

[8] Gurdon C., Svab Z., Feng Y., Kumar D., Maliga P., Cell-to-cell movement of mitochondria in plants. Proc. Natl. Acad. Sci. U.S.A. 113, 3395–3400 (2016). 26951647 10.1073/pnas.1518644113 PMC4812711

[9] Bock R., Witnessing genome evolution: experimental reconstruction of endosymbiotic and horizontal gene transfer. Annu. Rev. Genet. 51, 1–22 (2017). 28846455 10.1146/annurev-genet-120215-035329

[10] Bock D. G., Andrew R. L., Rieseberg L. H., On the adaptive value of cytoplasmic genomes in plants. Mol. Ecol. 23, 4899–4911 (2014). 25223488 10.1111/mec.12920

[11] Bruun-Lund S., Clement W. L., Kjellberg F., Ronsted N., First plastid phylogenomic study reveals potential cyto-nuclear discordance in the evolutionary history of *Ficus L.* (Moraceae). Mol. Phylogenet. Evol. 109, 93–104 (2017). 28042043 10.1016/j.ympev.2016.12.031

[12] Alwadani K. G., Janes J. K., Andrew R. L., Chloroplast genome analysis of box-ironbark Eucalyptus. Mol. Phylogenet. Evol. 136, 76–86 (2019). 30954587 10.1016/j.ympev.2019.04.001

[13] Bock R., The give-and-take of DNA: horizontal gene transfer in plants. Trends Plant Sci. 15, 11–22 (2010). 19910236 10.1016/j.tplants.2009.10.001

[14] Rebbeck C. A., Leroi A. M., Burt A., Mitochondrial capture by a transmissible cancer. Science 331, 303 (2011). 21252340 10.1126/science.1197696

[15] Tan A. S., Baty J. W., Dong L.-D., Bezawork-Geleta A., Endaya B., Goodwin J., Bajzikova M., Kovarova J., Peterka M., Yan B., Alizadeh Pesdar E. A., Sobol M., Filimonenko A., Stuart S., Vondrusova M., Kluckova K., Sachaphibulkij K., Rohlena J., Hozak P., Truksa J., Eccles D., Haupt L. M., Griffiths L. R., Neuzil J., Berridge M. V., Mitochondrial genome acquisition restores respiratory function and tumorigenic potential of cancer cells without mitochondrial DNA. Cell Metab. 21, 81–94 (2015). 25565207 10.1016/j.cmet.2014.12.003

[16] Dong L.-F., Kovarova J., Bajzikova M., Bezawork-Geleta A., Svec D., Endaya B., Sachaphibulkij K., Coelho A. R., Sebkova N., Ruzickova A., Tan A. S., Kluckova K., Judasova K., Zamecnikova K., Rychtarcikova Z., Gopalan V., Andera L., Sobol M., Yan B., Pattnaik B., Bhatraju N., Truksa J., Stopka P., Hozak P., Lam A. K., Sedlacek R., Oliveira P. J., Kubista M., Agrawal A., Dvorakova-Hortova K., Rohlena J., Berridge M. V., Neuzil J., Horizontal transfer of whole mitochondria restores tumorigenic potential in mitochondrial DNA-deficient cancer cells. eLife 6, e22187 (2017). 28195532 10.7554/eLife.22187 PMC5367896

[17] Hayakawa K., Esposito E., Wang X., Terasaki Y., Liu Y., Xing C., Ji X., Lo E. H., Transfer of mitochondria from astrocytes to neurons after stroke. Nature 535, 551–555 (2016). 27466127 10.1038/nature18928 PMC4968589

[18] Stegemann S., Keuthe M., Greiner S., Bock R., Horizontal transfer of chloroplast genomes between plant species. Proc. Natl. Acad. Sci. U.S.A. 109, 2434–2438 (2012). 22308367 10.1073/pnas.1114076109 PMC3289295

[19] Lu Y., Stegemann S., Agrawal S., Karcher D., Ruf S., Bock R., Horizontal transfer of a synthetic metabolic pathway between plant species. Curr. Biol. 27, 3034–3041.e3 (2017). 28943084 10.1016/j.cub.2017.08.044

[20] Thyssen G., Svab Z., Maliga P., Cell-to-cell movement of plastids in plants. Proc. Natl. Acad. Sci. U.S.A. 109, 2439–2443 (2012). 22308369 10.1073/pnas.1114297109 PMC3289365

[21] Oparka K. J., Roberts A. G., Boevink P., Santa Cruz S., Roberts I., Pradel K. S., Imlau A., Kotlizky G., Sauer N., Epel B., Simple, but not branched, plasmodesmata allow the nonspecific trafficking of proteins in developing tobacco leaves. Cell 97, 743–754 (1999). 10380926 10.1016/s0092-8674(00)80786-2

[22] Kim I., Cho E., Crawford K., Hempel F. D., Zambryski P. C., Cell-to-cell movement of GFP during embryogenesis and early seedling development in *Arabidopsis*. Proc. Natl. Acad. Sci. U.S.A. 102, 2227–2231 (2005). 15668382 10.1073/pnas.0409193102 PMC548566

[23] Jeffree C. E., Yeoman M. M., Development of intercellular connections between opposing cells in a graft union. New Phytol. 93, 491–509 (1983).

[24] Melnyk C. W., Schuster C., Leyser O., Meyerowitz E. M., A developmental framework for graft formation and vascular reconnection in *Arabidopsis thaliana*. Curr. Biol. 25, 1306–1318 (2015). 25891401 10.1016/j.cub.2015.03.032 PMC4798781

[25] Sidorov V., Armstrong C., Ream T., Ye X., Saltarikos A., “Cell grafting”: A new approach for transferring cytoplasmic or nuclear genome between plants. Plant Cell Rep. 37, 1077–1089 (2018). 29779094 10.1007/s00299-018-2292-7

[26] Ehlers K., Kollmann R., Primary and secondary plasmodesmata: structure, origin, and functioning. Protoplasma 216, 1–30 (2001). 11732191 10.1007/BF02680127

[27] Chiba A., Ishida H., Nishizawa N. K., Makino A., Mae T., Exclusion of ribulose-1,5-bisphosphate carboxylase/oxygenase from chloroplasts by specific bodies in naturally senescing leaves of wheat. Plant Cell Physiol. 44, 914–921 (2003). 14519773 10.1093/pcp/pcg118

[28] Ishida H., Izumi M., Wada S., Makino A., Roles of autophagy in chloroplast recycling. Biochim. Biophys. Acta 1837, 512–521 (2014). 24269172 10.1016/j.bbabio.2013.11.009

[29] Köhler R. H., Cao J., Zipfel W. R., Webb W. W., Hanson M. R., Exchange of protein molecules through connections between higher plant plastids. Science 276, 2039–2042 (1997). 9197266 10.1126/science.276.5321.2039

[30] Köhler R. H., Hanson M. R., Plastid tubules of higher plants are tissue-specific and developmentally regulated. J. Cell Sci. 113, 81–89 (2000). 10591627 10.1242/jcs.113.1.81

[31] Gunning B. E. S., Plastid stromules: video microscopy of their outgrowth, retraction, tensioning, anchoring, branching, bridging, and tip-shedding. Protoplasma 225, 33–42 (2005). 15868211 10.1007/s00709-004-0073-3

[32] Yamane K., Mitsuya S., Taniguchi M., Miyake H., Salt-induced chloroplast protrusion is the process of exclusion of ribulose-1,5-bisphosphate carboxylase/oxygenase from chloroplasts into cytoplasm in leaves of rice. Plant Cell Environ. 35, 1663–1671 (2012). 22489666 10.1111/j.1365-3040.2012.02516.x

[33] Newell C. A., Natesan S. K. A., Sullivan J. A., Jouhet J., Kavanagh T. A., Gray J. C., Exclusion of plastid nucleoids and ribosomes from stromules in tobacco and *Arabidopsis*. Plant J. 69, 399–410 (2012). 21951134 10.1111/j.1365-313X.2011.04798.x

[34] Kragler F., Lucas W. J., Monzer J., Plasmodesmata: dynamics, domains and patterning. Ann. Bot. 81, 1–10 (1998).

[35] Cheong Y. H., Chang H.-S., Gupta R., Wang X., Zhu T., Luan S., Transcriptional profiling reveals novel interactions between wounding, pathogen, abiotic stress, and hormonal responses in *Arabidopsis*. Plant Physiol. 129, 661–677 (2002). 12068110 10.1104/pp.002857 PMC161692

[36] Nanda A. K., Melnyk C. W., The role of plant hormones during grafting. J. Plant Res. 131, 49–58 (2018). 29181647 10.1007/s10265-017-0994-5 PMC5762790

[37] Thomson W. W., Whatley J. M., Development of nongreen plastids. Annu. Rev. Plant Physiol. 31, 375–394 (1980).

[38] Itoh R. D., Fujiwara M. T., Regulation of leucoplast morphology in roots: Interorganellar signaling from mitochondria? Plant Signal. Behav. 5, 856–859 (2010). 20505352 10.4161/psb.5.7.11893 PMC3014538

[39] Natesan S. K. A., Sullivan J. A., Gray J. C., Stromules: a characteristic cell-specific feature of plastid morphology. J. Exp. Bot. 56, 787–797 (2005). 15699062 10.1093/jxb/eri088

[40] Sattarzadeh A., Krahmer J., Germain A. D., Hanson M. R., A myosin XI tail domain homologous to the yeast myosin vacuole-binding domain interacts with plastids and stromules in Nicotiana benthamiana. Mol. Plant 2, 1351–1358 (2009). 19995734 10.1093/mp/ssp094

[41] Natesan S. K. A., Sullivan J. A., Gray J. C., Myosin XI is required for actin-associated movement of plastid stromules. Mol. Plant 2, 1262–1272 (2009). 19995729 10.1093/mp/ssp078

[42] Wang J., Jiang L., Wu R., Plant grafting: how genetic exchange promotes vascular reconnection. New Phytol. 214, 56–65 (2017). 27991666 10.1111/nph.14383

[43] Davis C. C., Wurdack K. J., Host-to-parasite gene transfer in flowering plants: phylogenetic evidence from Malpighiales. Science 305, 676–678 (2004). 15256617 10.1126/science.1100671

[44] Mower J. P., Stefanovic S., Young G. J., Palmer J. D., Gene transfer from parasitic to host plants. Nature 432, 165–166 (2004). 10.1038/432165b 15538356

[45] Melnyk C. W., Meyerowitz E. M., Plant grafting. Curr. Biol. 25, R183–R188 (2015). 25734263 10.1016/j.cub.2015.01.029

[46] Murashige T., Skoog F., A revised medium for rapid growth and bio assays with tobacco tissue culture. Physiol. Plant. 15, 473–497 (1962).

[47] Svab Z., Maliga P., High-frequency plastid transformation in tobacco by selection for a chimeric aadA gene. Proc. Natl. Acad. Sci. U.S.A. 90, 913–917 (1993). 8381537 10.1073/pnas.90.3.913 PMC45780

