# Supplementary material for: Horizontal genome transfer by cell-to-cell travel of whole organelles
Source: Sci Adv. 2021 Jan 1;7(1):eabd8215. doi: 10.1126/sciadv.abd8215 (PMC7775762; doi:10.1126/sciadv.abd8215)
Supplement: http://advances.sciencemag.org/cgi/content/full/7/1/eabd8215/DC1 [file supp_7_1_eabd8215__index.html]

Science Advances | Science AdvancesAAASSearchScience AdvancesMenu

## Supplementary Materials

# Horizontal genome transfer by cell-to-cell travel of whole organelles

Alexander P. Hertle, Benedikt Haberl, Ralph Bock

Download Supplement

**The PDF file includes:**

- Table S1
- Figs. S1 to S4
- Legends for videos S1 to S5

**Other Supplementary Material for this manuscript includes the following:**

- Video S1
- Video S2
- Video S3
- Video S4
- Video S5

**Files in this Data Supplement:**

- Adobe PDF - abd8215\_SM.pdf
- abd8215\_Video\_S1.mp4
- abd8215\_Video\_S2.mp4
- abd8215\_Video\_S3.mp4
- abd8215\_Video\_S4.mp4
- abd8215\_Video\_S5.mp4
