# Supplementary material for: Horizontal genome transfer by cell-to-cell travel of whole organelles
Source: Sci Adv. 2021 Jan 1;7(1):eabd8215. doi: 10.1126/sciadv.abd8215 (PMC7775762; doi:10.1126/sciadv.abd8215)
Supplement: http://advances.sciencemag.org/cgi/content/full/7/1/eabd8215/DC1 [file supp_7_1_eabd8215__1.pdf]

Science Advances
AAAS

advances.sciencemag.org/cgi/content/full/7/1/eabd8215/DC1

Supplementary Materials for

Horizontal genome transfer by cell-to-cell travel of whole organelles

Alexander P. Hertle, Benedikt Haberl, Ralph Bock*

*Corresponding author. Email: rbock@mpimp-golm.mpg.de

Published 1 January 2021, *Sci. Adv.* **7**, eabd8215 (2021)
DOI: 10.1126/sciadv.abd8215

The PDF file includes:

Table S1
Figs. S1 to S4
Legends for videos S1 to S5

Other Supplementary Material for this manuscript includes the following:

(available at advances.sciencemag.org/cgi/content/full/7/1/eabd8215/DC1)

Videos S1 to S5

SUPPLEMENTARY MATERIALS

Supplementary table S1. Measurement of plastid sizes. Values represent average diameters +/- standard deviation in µm.

**Figure 1B**	Type III	Type IV
Plastids in recipient cell	6.77 µm +/- 1.71	4.5 µm +/- 1.41
Transferred plastids	2.1 µm +/- 0.3	3.2 µm +/- 1.03
Plastids in donor cell	4.99 µm +/-2.4	7.5 µm +/- 0.98

			Lower Panel	
**Figure 2C**	Top Panel	Middle Panel	I	II
Plastids in recipient cell	5.95 µm +/-1.7	6.19 µm +/- 1.96	5.48 µm +/- 1.61	6.03 µm +/- 1.79
Transferred plastids	2.7 µm +/- 1	2.01 µm +/- 0.26	4.62 µm +/- 1.56	2.8 µm +/- 0.77
Plastids in donor cell	4 µm +/-1.3	4.83 µm +/- 1.1	4.89 µm +/- 0.75	3.91 µm +/- 1.35

**Figure 4A**	
Plastids in recipient cell	5.54 µm +/-1
Transferred plastids	2.26 µm
Plastids in donor cell	5.23 µm +/-0.68

**Figure 6B**	
Plastids in recipient cell	2.52 µm +/-1.4
transferred plastids	2.01 µm +/-0.29
Plastids in donor cell	3.55 m +/-1.47

**Figure S4A**	
Dark, no sucrose	2.12 µm +/-0.69
Light + sucrose	5.56 µm +/-1.45

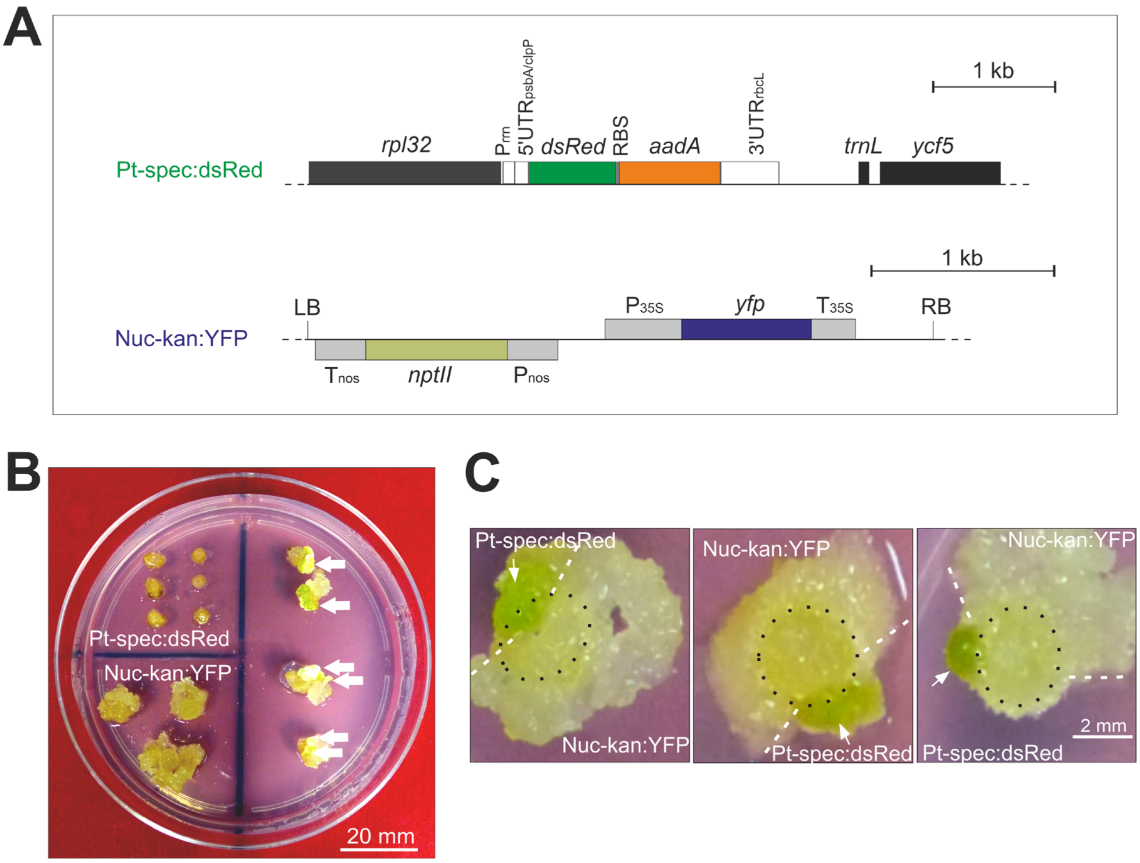

Supplementary fig. S1. Genetic selection for horizontal transfer of plastid genomes across graft junctions. (**A**) Transgenic constructs used to visualize horizontal movement of plastid genomes. Pt-spec:dsRed plants possess a spectinomycin resistance gene (*aadA*) and the gene for the red fluorescent protein dsRed stably integrated into their plastid genome. Nuc-kan:YFP plants harbor a kanamycin resistance gene (*nptII*) and the gene for the yellow fluorescent protein YFP in their nuclear genome. P_{rrn}: chloroplast rRNA operon promoter; $5'UTR_{psbA/clpP}$: chimeric 5'UTR from the chloroplast *psbA* and *clpP* genes; RBS: synthetic Shine-Dalgarno sequence (ribosome-binding site); $3'UTR_{rbcL}$: 3' UTR from the chloroplast *rbcL* gene of *Chlamydomonas reinhardtii*; P_{35S}: CaMV 35S promoter; T_{35S}: CaMV 35S terminator; P_{nos}: nopaline synthase gene promoter from *Agrobacterium tumefaciens*; T_{nos}: nopaline synthase

terminator. (**B**) Visualization of horizontal genome transfer by exposure of excised graft sites to double selection for kanamycin and spectinomycin (6, 18, 19). Plastid genome transfer events are evidenced by callus growth and plant regeneration in the presence of both kanamycin and spectinomycin (indicated by white arrows). As a control, stem sections distant from the graft site were subjected to double selection on the same medium (left half of the Petri dish). Photo credit: B. Haberl, Max-Planck-Institut für Molekulare Pflanzenphysiologie. (**C**) Close-up of three independent events of genome transfer (identified as callus tissue growing in the presence of both antibiotics; white arrows). Doubly resistant calli always originate from the fused cambial rings of the graft union (black dotted line). The white dashed lines mark the borders between scion and stock. Photo credit: B. Haberl, Max-Planck-Institut für Molekulare Pflanzenphysiologie.

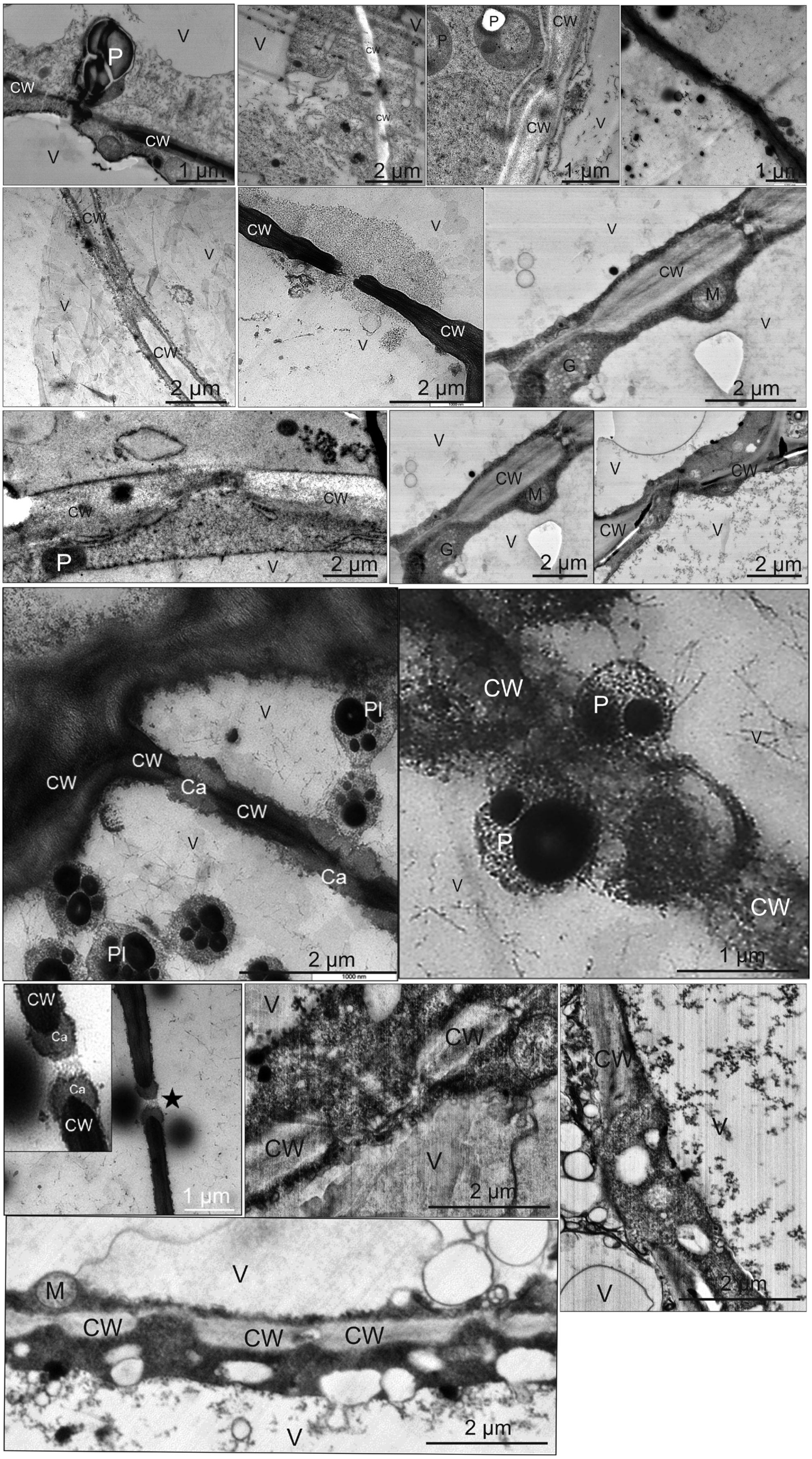

P
V
CW
V
1 µm
V
CW
CW
2 µm
P
P
CW
CW
V
1 µm
1 µm
CW
V
V
CW
2 µm
CW
V
V
CW
2 µm
V
CW
M
V
G
2 µm
CW
CW
P
V
2 µm
V
CW
M
G
V
2 µm
V
CW
CW
V
2 µm
V
Pl
CW
CW
Ca
CW
V
Ca
Pl
2 µm
CW
P
V
P
V
CW
1 µm
CW
Ca
Ca
CW
★
1 µm
V
CW
CW
V
2 µm
CW
V
V
2 µm
M
V
CW
CW
CW
V
2 µm

Supplementary fig. S2. Establishment of intercellular connections between callus cells in graft unions. The collection of electron microscopic images illustrates cell wall thinning and formation of pores between callus cells at the graft junction. It also shows the frequent presence of DNA-containing organelles (plastids and mitochondria) near or within the pores. CW: cell wall, V: vacuole, P: plastid, M: mitochondrion, G: Golgi apparatus, Ca: callose.

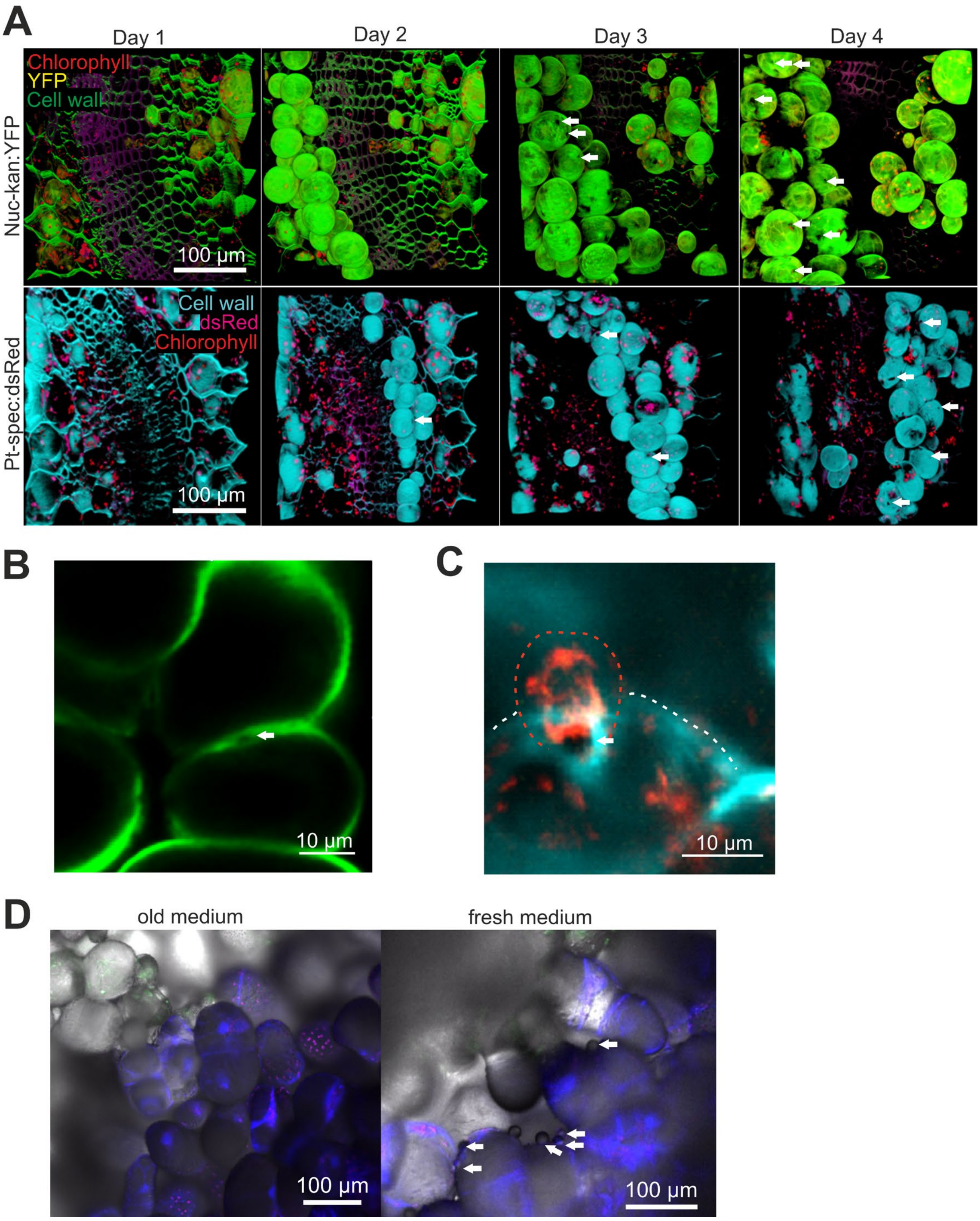

Supplementary fig. S3. Changes in cell wall surface and callus cell budding. (A) Hole formation in the cell wall of proliferating callus cells at the graft junction. Changes in cell wall structure (of Pt-spec:dsRed and Nuc-kan:YFP cells) were analyzed over several days after grafting by live cell confocal microscopy. Prior to imaging, cell walls

were stained with calcofluor white. After two days, the first callus cells appeared at the cambial ring of the cut stem surface. The cells showed homogeneous staining of the cell wall surface and no pores. At day 3 and 4, holes started to appear within the cell wall. For Nuc-kan:YFP cells, chlorophyll fluorescence is shown in red, YFP fluorescence in yellow and calcofluor fluorescence in green. For Pt-spec:dsRed, chlorophyll fluorescence is shown in red, dsRed fluorescence in magenta and calcofluor fluorescence in cyan. (**B**) Cell wall pores in callus cells. In addition to pores in surface-exposed walls of callus cells, intercellular pores connecting appressed callus cells three days after grafting were also detected. (**C**) Plastids emerging through the cell wall pores. Cell walls were stained with calcofluor white (cyan), and the white dashed line indicates the cell wall periphery. Plastids (red) emerge through a cell wall opening within a bud-like protrusion (red dashed line). (**D**) Budding of Nuc-kan:YFP callus cells. Following transfer of callus tissue to fresh growth medium, the number of bud-like protrusions on the cell surface increased. The cytosolically localized YFP (blue) is detectable inside several buds.

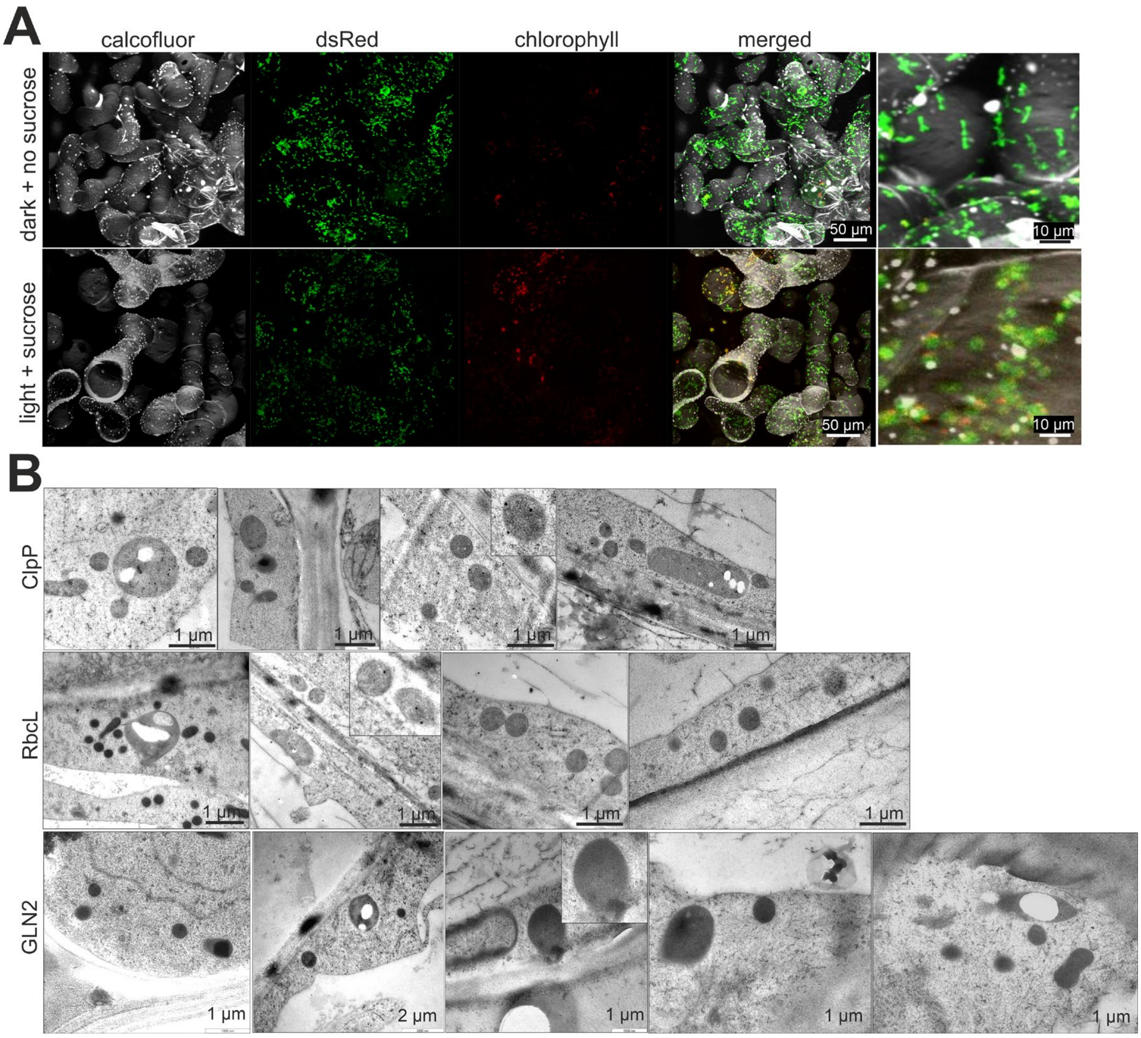

Supplementary fig. S4. Plastid dedifferentiation and presence of DNA in dedifferentiated plastids. (**A**) After three days in the dark in the absence of an external carbon source, plastid have undergone a dramatic size reduction. Note that the size reduction occurs prior to the loss of chlorophyll fluorescence. (**B**) Identification of dedifferentiated organelles as plastids by immuno-electron microscopy. In order to identify the small organelles observed in callus cells at graft junctions, plastid proteins were immuno-localized with specific antibodies. By using anti-ClpP and anti-GLN2 antibodies (recognizing a subunit of the stromal Clp protease and the plastid glutamine synthetase, respectively), the organelles were clearly identified as plastids. Anti-RbcL

antibodies (recognizing the large subunit of Rubisco) also labeled the organelles, although this antibody also shows some non-specific signals outside of plastids.

Supplementary Video S1. Limited mobility of plastids in the presence of light and sucrose as an external source of reduced carbon.

Supplementary Video S2. Increased mobility of plastids under starvation conditions (incubation of callus tissue in darkness an in the absence of an external carbon source).

Supplementary Video S3. Cell-to-cell transfer of a plastid in a graft union observed in real time (cf. Fig. 8B, upper panel).

Supplementary Video S4. Cell-to-cell transfer of a plastid in a graft union observed in real time (cf. Fig. 8B, middle panel).

Supplementary Video S5. Cell-to-cell transfer of a plastid through a cell wall pore observed in real time (cf. Fig. 8B, bottom panel).
